# A comparative evaluation of five phenotypic methods for identification of carbapenemase-producing Enterobacteriaceae: a modified carbapenemase detection test

**DOI:** 10.1128/spectrum.00386-24

**Published:** 2024-06-04

**Authors:** Hamid Solgi, Ali Badamchi, Fereshteh Shahcheraghi, Farzad Badmasti, Mojtaba Akbari, Mehdi Behzadfar

**Affiliations:** 1 Isfahan Endocrine and Metabolism Research Center, Isfahan University of Medical Sciences, Isfahan, Iran; 2 Department of Laboratory Medicine, Amin Hospital, Isfahan University of Medical Sciences, Isfahan, Iran; 3 Childrens Medical Center, Tehran University of Medical Sciences, Tehran, Iran; 4 Department of Bacteriology, Pasteur Institute of Iran, Tehran, Iran; MultiCare Health System, Tacoma, Washington, USA

**Keywords:** carbapenemase, phenotypic detection, Enterobacteriaceae

## Abstract

Rapid detection of carbapenemase-producing Enterobacteriaceae (CPE) is urgently needed to prevent their spread in healthcare settings. Here, we have evaluated the performance of the phenotypic methods for detection of carbapenemase production directly from bacterial cultures. A total of 99 clinical and rectal Enterobacteriaceae isolates were included (81 carrying known carbapenemase-encoding genes and 18 without carbapenemase production). All isolates were subjected to the five phenotypic tests including in-house Carba NP (iCarba NP), modified-Carba NP, E-Test MBL, modified Hodge test (MHT), and commercial combination disk test. Test results were read at different time points for iCarba NP and modified-Carba (1 min, 5 min, 15 min, 1 h and 2 h). The sensitivity and specificity of the iCarba NP were 78.87% and 100%, respectively, whereas those of the modified-Carba NP test were 95.06% and 94.44%, respectively. False-negative results were detected in four OXA-48 isolates with the use of modified-Carba NP, whereas one non-carbapenemase isolate had false-positive results. The sensitivity/specificity was 91.30%/100% and 80.25%/83.33% for the E-Test MBL and MHT, respectively. The sensitivity and specificity of the aminophenylboronic acid synergy test were 100% and 97.94%, respectively, whereas those of the dipicolinic acid synergy test were 82.61% and 96.23%, respectively. Rapid, simple, and reliable methods are needed for laboratory detection of CPE isolates to improve the detection and surveillance of these clinically relevant pathogens in an epidemiological context. We conclude that the modified-Carba NP test can be one of the reliable tests for the prediction of carbapenemase-producing bacteria.

**IMPORTANCE:**

The emergence of carbapenem resistance among Gram-negative bacteria is a serious global health threat. Here, we investigate the performance of the five phenotypic assays against carbapenemase-producing and carbapenemase-non-producing Enterobacteriaceae. Accurate and rapid detection of CPE isolates is critically required for clinical management and treatment of infections caused by these organisms. Among the five evaluated phenotypic tests, the mCNP test presented the highest sensitivity (95.06%) and, therefore, can be considered the best test to be used as a screening phenotypic methodology.

## INTRODUCTION

The continuous worldwide spread of carbapenem-resistant Enterobacteriaceae (CRE) is an issue of great clinical and public health concern due to the limited therapeutic options available against infections caused by these organisms (1). The most common mechanism of carbapenem resistance is the production of carbapenem-hydrolyzing β-lactamases (carbapenemase-producing CRE [CPE]). However, other mechanisms contribute to carbapenem resistance, such as overexpression of AmpC, porin loss, and efﬂux pumps. Three main groups of enzymes are responsible for most carbapenem resistance: Ambler class A (KPC), B (Verona Integron-encoded MBL [VIM], New Delhi metallo-β-lactamase-1 [NDM-1], and IMP), and D (OXA-48-like) (2, 3). The rapid and accurate detection of CPE is essential for infection control purposes, especially in nosocomial outbreaks, and may help improve patient management and clinical outcome. Although rapid polymerase chain reaction (PCR) tests for the detection of the major carbapenemase gene families, phenotypic detection may be indicated when molecular methods are not readily available (4). Currently, a number of phenotypic tests have been described for phenotypic detection of CPE in many laboratories, including growth-based assays (combined disc test [CDT], modified Hodge test [MHT], and carbapenem inactivation method [mCIM]) and rapid biochemical assays (e.g., Carba NP and Blue-Carba methods) (2, 3, 5–7). Recently, the Clinical & Laboratory Standards Institute (CLSI) proposed the use of Carba NP and mCIM assays for detecting carbapenemase on Enterobacteriaceae. Here, we investigated the performance of the five phenotypic assays against carbapenemase-producing and carbapenemase-non-producing Enterobacteriaceae. It should be noted that the Modified Carba NP test is a novel method for the rapid detection of carbapenemase activity.

## MATERIALS AND METHODS

### Bacterial strains

A total of 99 non-duplicated CRE isolates were used in this study. These isolates were collected between 2015 and 2018 from various clinical samples and surveillance samples of two Iranian University hospitals (Tehran and Isfahan). All isolates were genotyped with the same protocol. The test strains included 2 KPC producers (2 *Klebsiella pneumoniae*), 15 NDM producers (9 *K*. *pneumoniae*, 4 *Escherichia coli*, and 2 *Enterobacter cloacae*), 33 OXA-48-like producers (18 *K*. *pneumoniae*, 10 *E. coli*, 4 *Serratia marcescens*, and 1 *Proteus mirabilis*), 28 NDM-type and OXA-48 co-producers (6 *E. coli* and 22 *K*. *pneumoniae*), and 3 VIM-1 producers (3 *K*. *pneumoniae*). Furthermore, 18 non-carbapenemase-producing isolates (ESBL producers) were also included. All isolates were characterized for the presence of *bla*_KPC_, *bla*_GES_, *bla*_VIM_, *bla*_IMP_, *bla*_NDM_, *bla*_OXA-48_, and ESBL genes by PCR sequencing (8–11).

### Quality control

*K. pneumoniae* ATCC BAA 1705 (KPC-2), *K. pneumoniae* ATCC BAA 2146 (NDM-1), *E. coli* ATCC 25922, and *Pseudomonas aeruginosa* ATCC 27853 were used as controls.

### Phenotypic detection of carbapenemase production

All tested isolates were stored in nutrient broth (Difco, Detroit, MI, USA) containing 20% glycerol at −80°C and subcultured twice using 5% sheep blood agar at 35°C for 18–24 h before testing by the five phenotypic tests.

### Modified Hodge test

The MHT was performed as previously described, with a 10 µg imipenem or ertapenem disk (12).

### E-Test MBL

The E-Test MBL was performed based on manufacturer’s instructions. MIC Test Strip MBL strips consisting of imipenem (IMI)/imipenem + EDTA (IMD) or meropenem (MRP)/meropenem + EDTA (MRD) are designed to detect metallo-β-lactamases (MBL). The presence of MBL is indicated by a reduction of the IMI or MRP value by ≥3 log dilutions in the presence of EDTA or the appearance of a phantom zone or deformation of the IMI or MRP ellipse.

### Commercial combination disk assay

Commercial diagnostic disks (Liofilchem, Rosetodegli Abruzzi, Italy) containing meropenem (10 µg), meropenem+phenyl boronic acid (PBA) as a KPC inhibitor and meropenem+dipicolinic acid (DPA) as an MBL inhibitor were placed on Mueller–Hinton agar plates on which the test strains had been inoculated. Zone diameters were measured after overnight incubation at 37°C. An increase of ≥5 mm in the zone diameter around disks containing β-lactamase inhibitors, as compared with the disk with meropenem alone, was considered a positive result for PBA and DPA, according to the manufacturer’s instruction (6, 7).

### In-house carba NP test

**Fig 1:**
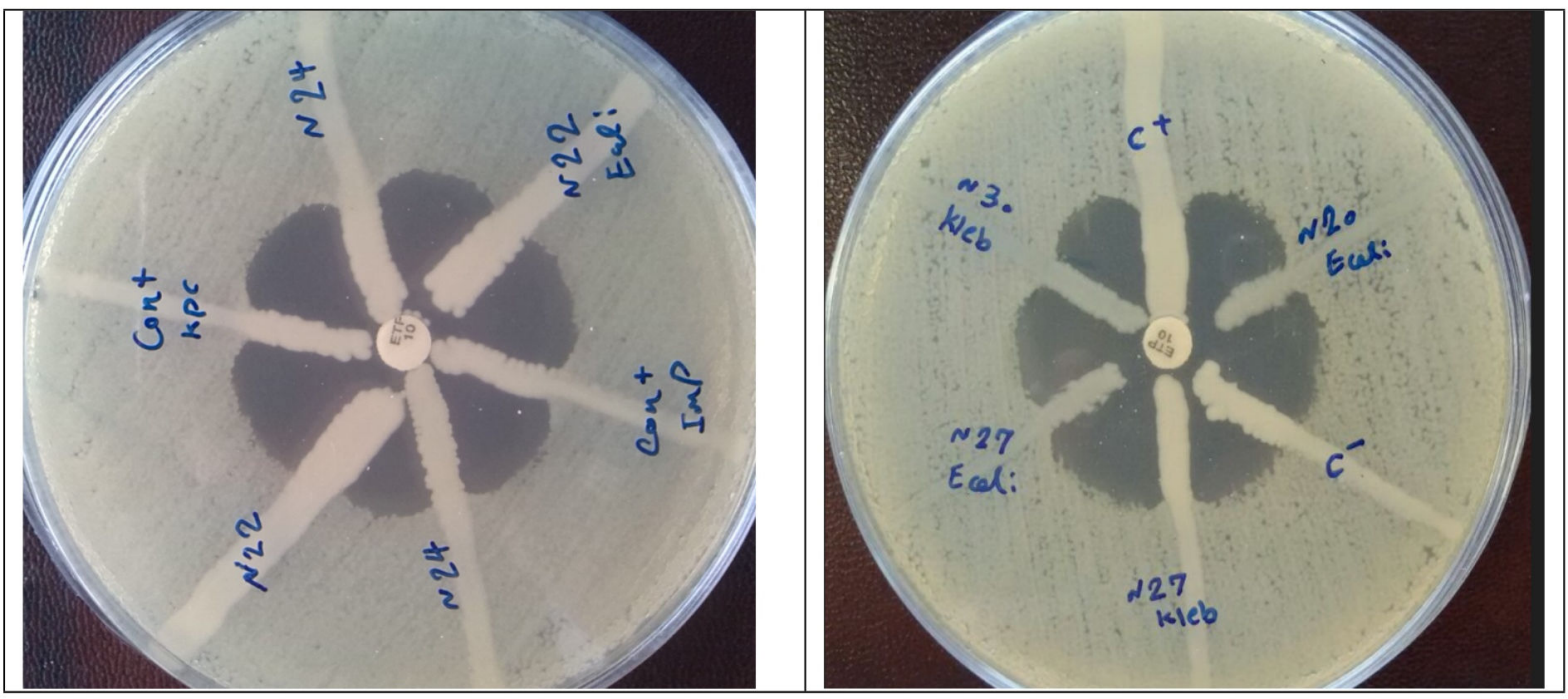
Examples of carbapenemase detection in reference strains and clinical isolates by the combination disk test.

The Carba-NP test was performed following the protocol recommended by CLSI (12). Brieﬂy, solution A (0.5% phenol red solution and 10 mM ZnSO4 solution, previously adjusted to pH 7.0) was prepared. On the day of the test, solution B, formed by solution A plus 10 mg/mL intravenous (i.v.) imipenem–cilastatin, was prepared. Paired 1.5-mL Eppendorf tubes were used for each isolate. The bacterial mass was scraped off with a calibrated loop (10 µL) and directly suspended in both tubes containing 100 µL of 20 mM Tris–HCl lysis buffer and was then vortexed for 5–10 s. Then, 100 µL of solution A was added, and the tube was vortexed again. The steps were repeated with 100 µL of solution B. The tubes were incubated at 37°C, and the results were examined every 15 min once for up to 2 h. Isolates giving any coloration change (yellow or light yellow) on the test strip (solution B) are interpreted as a positive result (Fig. 1). If the control strip turned yellow, the result was interpreted as invalid (Fig. 1).

### Modified carba NP test (mCNP)

Initially, 0.04% bromothymol blue and 0.04% neutral red (Merck Millipore, Germany) buffers were prepared. After pH adjustment (pH 7.0), the buffers were ﬁltered and mixed equally due to the number of test strains. Subsequently, a loop (10 µL) of a pure bacterial culture from Mueller–Hinton agar plates was resuspended in the 100-µL test mixture with (reaction tube) or without (control tube) supplementation with 5 mg/mL intravenous imipenem–cilastatin. Tubes were mixed for 5 to 10 seconds and incubated at 37°C and monitored for 2 h. Color changed from green or blue to yellow/orange in the antibiotic-containing tube, which was interpreted as a positive result. No change in initial green or blue was considered negative, whereas any color change in the control solution was treated as invalid. Strains were tested in duplicate.

### Statistical analysis

Statistical analysis of the phenotypic carbapenemase detection tests was conducted to evaluate the performance of each assay. Positive predictive value (PPV), negative predictive value (NPV), and kappa value were calculated to assess the reliability of the tests in identifying CPE isolates. Sensitivity and specificity were determined for the five phenotypic tests. These statistical measures provide insights into the accuracy and reliability of the phenotypic assays in detecting CPE isolates, contributing to the understanding of their utility in clinical settings. Statistical analyses were done using MedCalc ver. 12.2.1.0. (MedCalc Software, Ltd., Ostend, Belgium) and SPSS software for Windows (version 24, SPSS, Inc., Chicago, IL, USA).

## RESULTS

A total of 99 CRE isolates were selected for this study: 2 KPC, 15 NDM-type, 33 OXA-48, 28 NDM-type-OXA-48, 3 VIM producers, and 18 carbapenemase non-producers (Table 1). All the isolates were subjected to the five phenotypic assays. The performance results of the phenotypic carbapenemase detection tests are shown in Table 2.

**TABLE 1:** Results obtained for the five methods performed using a collection of carbapenem-resistant Enterobacteriaceae strains^*a*^

	Species (no. of strains with the same assay results)	iCarba NP	mCNP	E-Test MBL	MHT	CDT-DPA	CDT-PBA
Carbapenemase producer	*K. pneumoniae* (n = 54)						
Class A (n = 2) KPC-1		Pos (n = 2)	Pos (n = 2)	Pos (n = 0)	Pos (n = 2)	Pos (n = 0)	Pos (n = 2)
Class B (n = 12)							
VIM-1 (n = 3)		Pos (n = 3)	Pos (n = 3)	Pos (n = 3)	Pos (n = 2)	Pos (n = 3)	Pos (n = 0)
NDM-type (n = 9)		Pos (n = 9)	Pos (n = 9)	Pos (n = 9)	Pos (n = 5)	Pos (n = 7)	Pos (n = 2)
Class D (n = 18) OXA-48		Pos (n = 10)	Pos (n = 15)	Pos (n = 0)	Pos (n = 15)	Pos (n = 1)	Pos (n = 0)
Class B + class D (n = 22) NDM-type+OXA-type		Pos (n = 21)	Pos (n = 22)	Pos (n = 19)	Pos (n = 19)	Pos (n = 18)	Pos (n = 0)
	*E. coli* (n = 20)						
Class B (n = 4) NDM-1		Pos (n = 4)	Pos (n = 4)	Pos (n = 4)	Pos (n = 2)	Pos (n = 4)	Pos (n = 0)
Class D (n = 10) OXA-48		Pos (n = 5)	Pos (n = 9)	Pos (n = 0)	Pos (n = 10)	Pos (n = 0)	Pos (n = 0)
Class B + class D (n = 6) NDM-type+OXA-type		Pos (n = 5)	Pos (n = 5)	Pos (n = 5)	Pos (n = 4)	Pos (n = 4)	Pos (n = 0)
	*S. marcescens* (n = 4)						
Class D (n = 4) OXA-48		Pos (n = 4)	Pos (n = 4)	Pos (n = 0)	Pos (n = 4)	Pos (n = 0)	Pos (n = 0)
	*E. cloacae* (n = 2)						
Class B (n = 2) NDM-1		Pos (n = 2)	Pos (n = 2)	Pos (n = 2)	Pos (n = 1)	Pos (n = 2)	Pos (n = 0)
	*P. mirabilis* (n = 1)						
Class D (n = 1) OXA-48		Pos (n = 1)	Pos (n = 1)	Pos (n = 0)	Pos (n = 1)	Pos (n = 0)	Pos (n = 0)
Carbapenemase non-producer							
	*K. pneumoniae* (n = 16)	Pos (n = 0)	Pos (n = 1)	Pos (n = 0)	Pos (n = 3)	Pos (n = 1)	Pos (n = 0)
	*E. coli* (n = 2)	Pos (n = 0)	Pos (n = 0)	Pos (n = 0)	Pos (n = 0)	Pos (n = 0)	Pos (n = 0)

^*a*^ Pos, Positive; iCarba NP, in-house Carba NP; mCNP, modified Carba NP; MBL, metallo-β-lactamases; MHT, modified Hodge test; CDT DPA, Combination disk test dipicolinic acid; CDT-PBA, Combination disk test phenyl boronic acid.

**TABLE 2:** Performance characteristics of five phenotypic tests for detection of carbapenemase and noncarbapenemase-producing Enterobacteriaceae^*a*^

Test	Detection of β-lactamase	No. of positive isolates/no. of total isolates						Sensitivity	Speciﬁcity	PPV (95% CI)	NPV	Kappa
								(95% CI)	(95% CI)		(95% CI)	(SE)
		Carbapenemase producer					Carbapenemase non-producer					
			
		KPC-1	NDM-type	VIM-1	OXA-48	NDM-type-OXA-type	ESBL					
iCarba NP	KPC, MBL, OXA-48-like	2-Feb	15/15	3-Mar	20/33	26/28	0/18	78.87 (67.6–87. 7)	100.00 (81.5-100)	100.00 (93.6- 100)	54.55 (36.4–71.9)	0.602
mCNP	KPC, MBL, OXA-48-like	2-Feb	15/15	3-Mar	29/33	28/28	18-Jan	95.06 (87.8-98.6)	94.44 (72.7-99.8)	98.72 (93.1–99.9)	80.95 (58.1–94.6)	0.841 (0.069)
E-Test MBL	MBL	0/2	15/15	3-Mar	0/33	24/28	0/18	91.30 (79.2-97.6)	100.00 (93.3-100)	100	92.98 (83.0–98.1)	0.918 (0.040)
MHT	KPC, MBL, OXA-48-like	2-Feb	15-Aug	3-Feb	30/33	23/28	18-Mar	80.25 (69.9-88.3)	83.33 (58.6-96.4)	95.59 (87.6–99.1)	48.39 (30.2–66.9)	0.496 (0.095)
CDT-DPA	MBL	0/2	13/15	3-Mar	Jan-33	22/28	18-Jan	82.61 (68.6-92.2	96.23 (87.0-99.5)	95.00 (83.1–99.4)	86.44 (75.0–93.9)	0795 (0.061)
CDT-PBA	KPC	2-Feb	15-Feb	0/3	0/33	0/28	0/18	100.00 (15.8-100)	97.94 (92.7-99.8)	50.00 (6.8–93.2)	100.00 (96.2–100)	0.657 (0.225)

^*a*^ iCarba NP, In-house Carba NP; mCNP, modified Carba NP; MBL, metallo-β-lactamases; MHT, modified Hodge test; CDT-DPA, combination disk test-dipicolinic acid; CDT-PBA, combination disk test-phenyl boronic acid; CI, confidence interval; PPV, positive predictive value; NPV, negative predictive value; SE, standard error.

Using the MHT (Fig. 1), 16 of the 81 carbapenemase-producing isolates were negative, and 65 were positive. Of the 18 carbapenemase-negative isolates, three isolates were positive. The sensitivity and specificity of this test were calculated, respectively, 80.25% and 83.33%.

**Fig 2:**
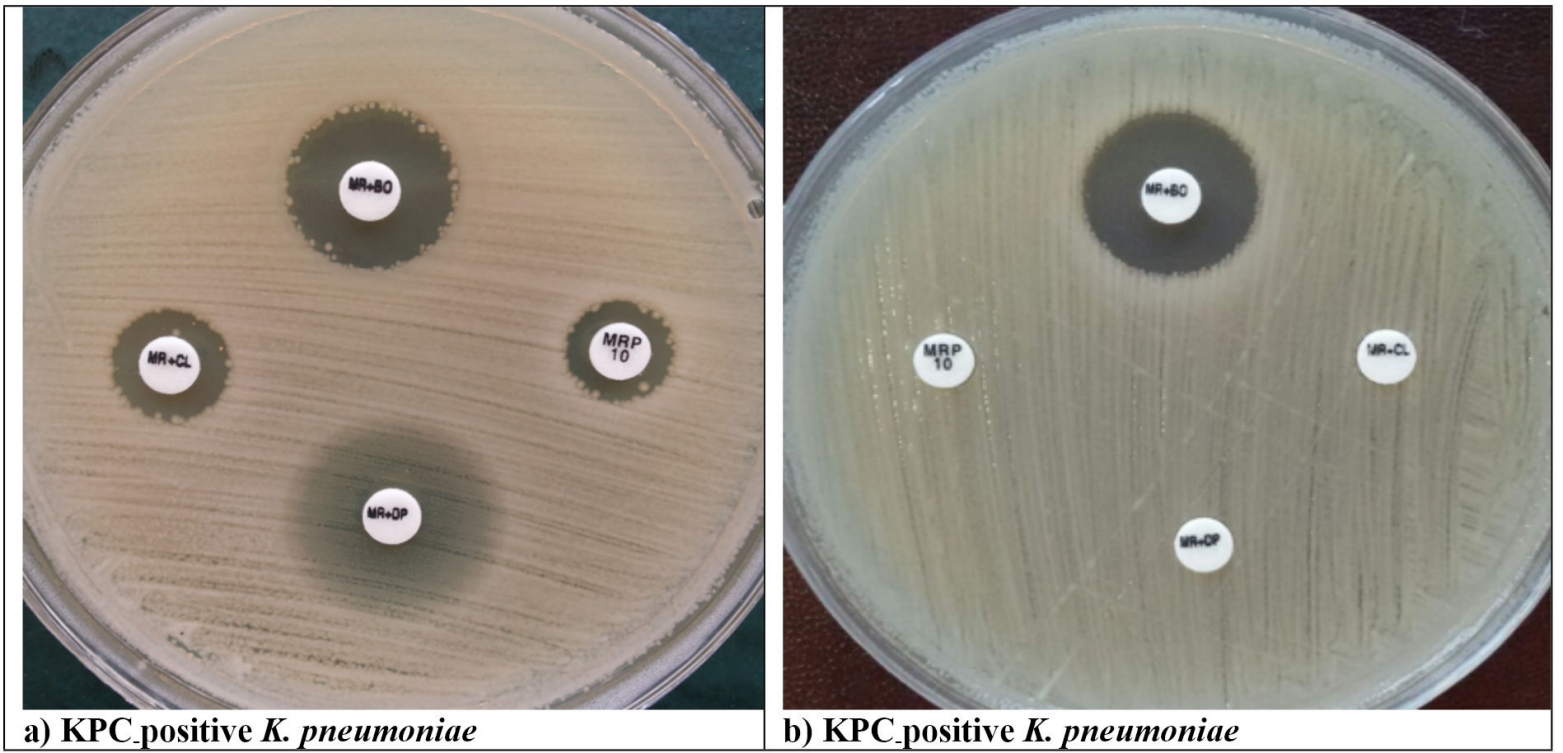
Examples of carbapenemase detection in reference strains and clinical isolates by using the modified Hodge test.

For identiﬁcation of KPC, the sensitivity of the PBA synergy test was 100%, with a speciﬁcity of 97.94%. Two isolates were false-positive in the PBA synergy test. For identiﬁcation of class B carbapenemases (NDM and VIM), the sensitivity of the DPA synergy test was 82.61%, with a speciﬁcity of 96.23%. Eight MBL producers were not detected: two NDM-producing *K. pneumoniae,* two *E. coli*, and four *K. pneumoniae* isolates producing both NDM-type and OXA-48 were negative. One ESBL-producing *K. pneumoniae* isolate was false-positive in the DPA test (Fig. 2).

**Fig 3:**
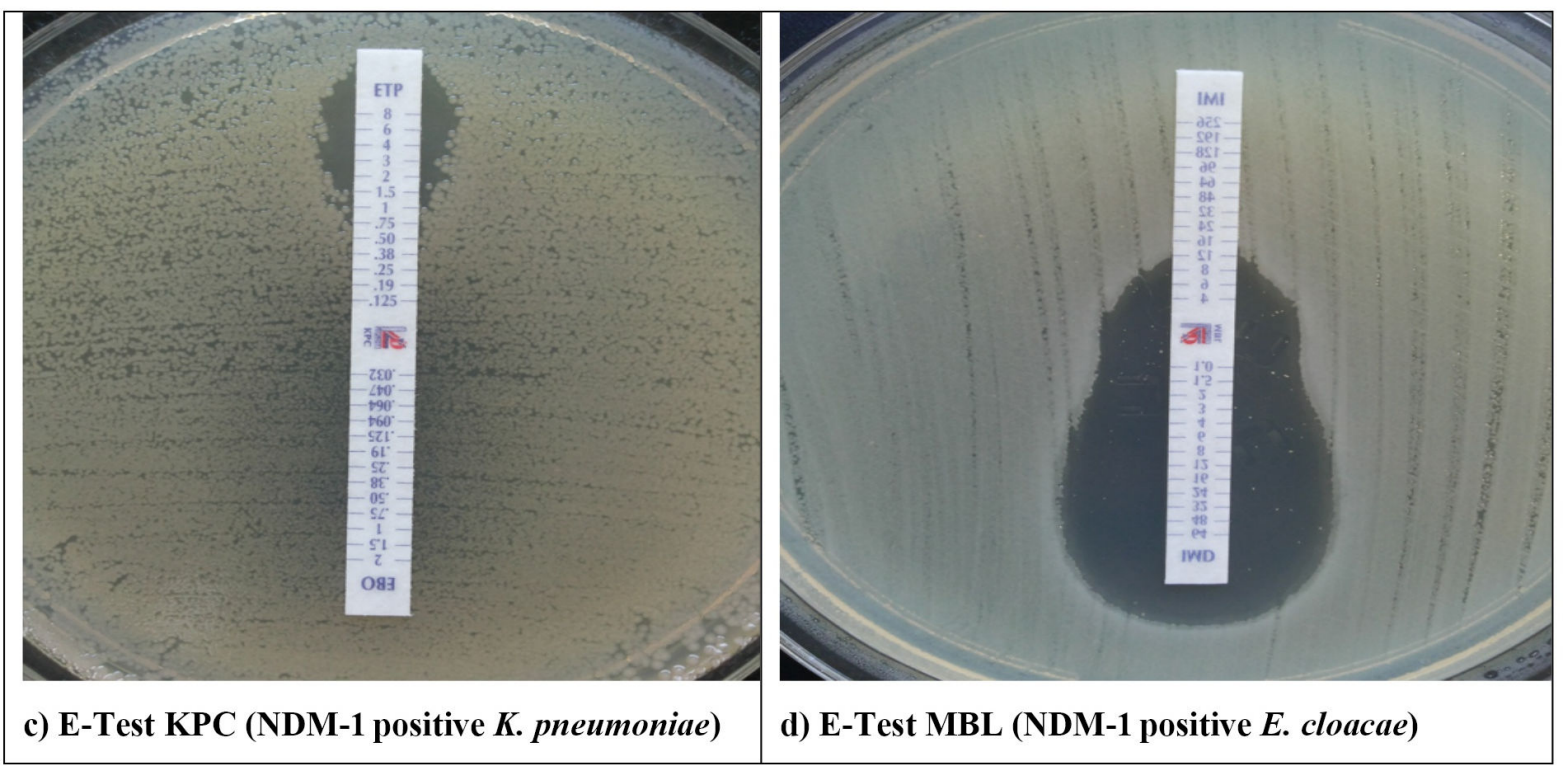
Examples of carbapenemase detection in reference strains and clinical isolates by using the E-Test.

Among the MBL producers, all 15 *bla*_NDM_ allele-positive and three *bla*_VIM_-1 positive isolates were detected with meropenem-containing E-test MBL (Fig. 3), whereas NDM type-OXA-48 co-producers were detected at 85.7% (24/28). The E-test MBL strip showed a high sensitivity of 91.3% and a specificity of 100%.

### In-house carba NP test

**Fig 4:**
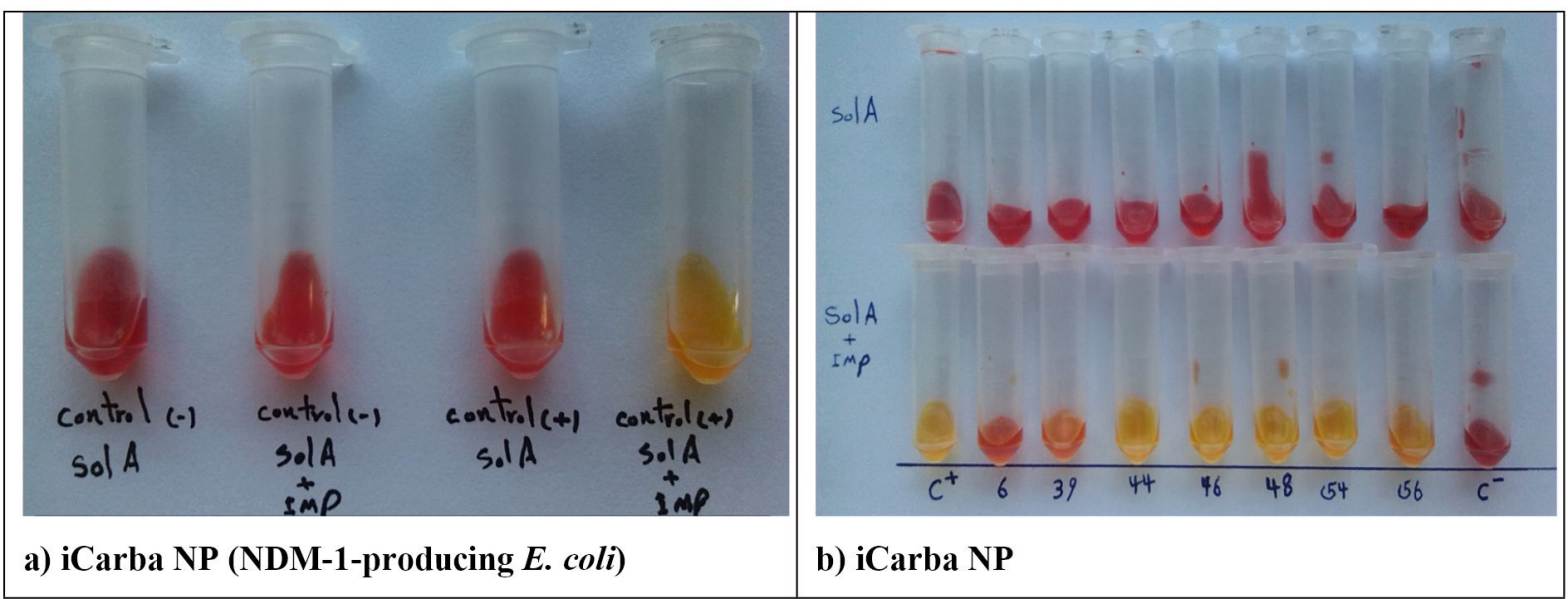
(a). iCarba NP test results at 2 h of incubation. Isolates were tested in paired tubes. The first and third tubes contain solution A: phenol red 0.05% + SO4 Zn 10 Mm, pH = 7.0. The second and fourth tubes contain solution A + 10 mg/mL imipenem–cilastatin. (C^-^) *E. coli* ATCC 25922. (C^+^) NDM-1-positive *E. coli*. (b). iCarba NP test results at 2 h of incubation. Isolates were tested in paired tubes. The top tubes contain solution A: phenol red 0.05% + SO4 Zn 10 Mm, pH = 7.0. The bottom tubes contain solution A + 10 mg/mL imipenem–cilastatin. (C^+^) NDM-1-positive *K. pneumoniae* ATCC BAA 2146. (6) Carbapenemase-negative *K. pneumoniae*. (39) OXA-48-positive *S. marcescens*. (44) NDM-1+OXA-181-positive *E. coli*. (46) NDM-7+OXA-48-positive *K. pneumoniae*. (48) NDM-1-positive *Enterobacter Cloacae*. (54) NDM-7 positive *K. pneumoniae*. (56) VIM-positive *K. pneumoniae*. (C^-^) *E. coli* ATCC 25922.

Different times were required to observe a positive result for different carbapenemases types. This test required a maximum of 2 h for monitoring color change, and it detected all KPC, most NDM, and VIM carbapenemases in 2–15 min, even without incubation in most cases. However, it took 2 h of incubation for the detection of most OXA-48 carbapenemase (Fig. 4). Overall, 15 CPE isolates yielded false-negative or invalid results with in-house Carba NP test. The type of carbapenemase in the 15 isolates included 2 NDM-type OXA-48 and 13 OXA-48. All non-carbapenemase producers (ESBL) gave negative results. The sensitivity and specificity of the method were 78.87% and 100%, respectively.

### Modified carba NP test

**Fig 5:**
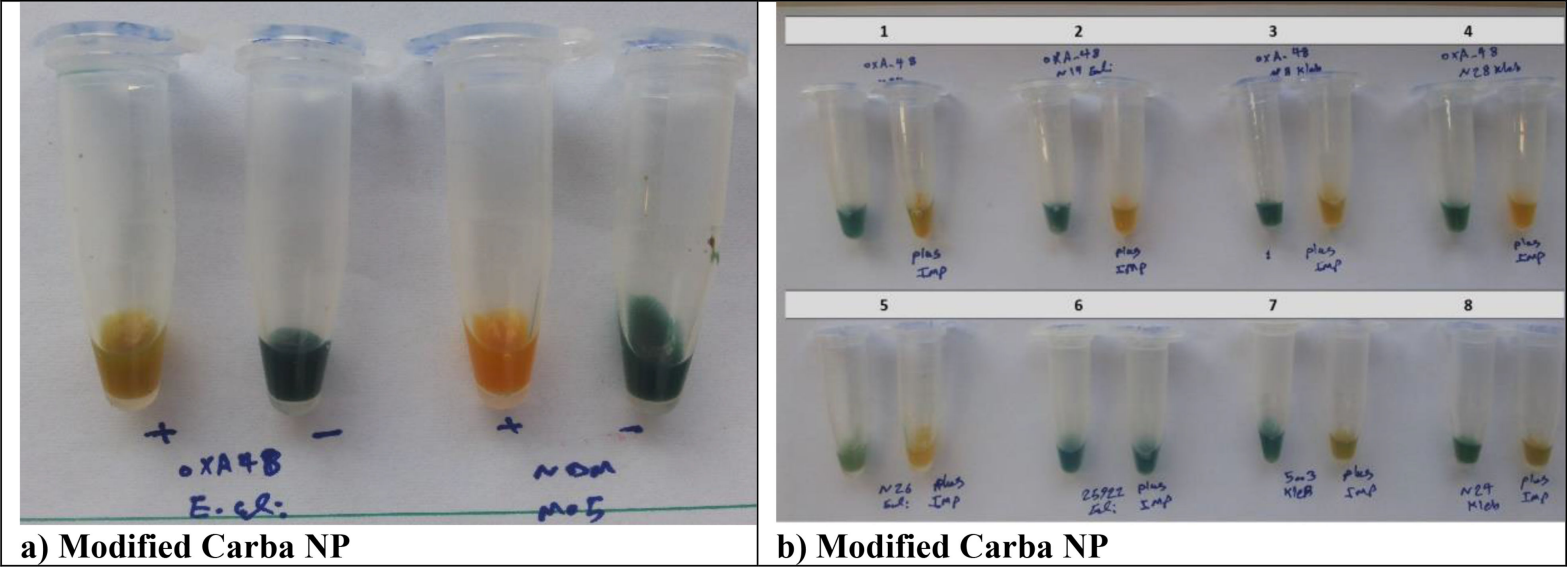
(a). Modified Carba NP test results at 2 h of incubation. Isolates were tested in paired tubes. The right tubes contain solution A: 0.04% bromothymol blue and 0.04% neutral red, pH = 7.0. The left tubes contain solution A + 5 mg/mL imipenem–cilastatin. Two left tubes, OXA-48-positive *E. coli*; two right tubes, NDM-1-positive *E. cloacae*. (b). Modified Carba NP test results at 2 h of incubation. Isolates were tested in paired tubes. The right tubes contain solution A: 0.04% bromothymol blue and 0.04% neutral red, pH = 7.0. The left tubes contain solution A + 5 mg/mL imipenem–cilastatin. (1) OXA-48-positive *S. marcescens*. (2) OXA-48-positive *E. coli* (3 and 4) and OXA-48-positive *K. pneumoniae*. (5) NDM-1-positive *E. coli*. (6) *E. coli* ATCC 25922. (7) KPC-positive *K. pneumoniae*. (8) NDM-1+OXA-48-positive *K. pneumoniae*.

Using the mCNP test, most carbapenemases were detected (Table 2). The vast majority of isolates with positive results presented a clear color change within 15 min of incubation; only OXA-48 producers required the maximum time of 2 h to become positive (Fig. 5). False-negative results occurred with four OXA-48 producers, including one *E. coli* and three *K. pneumonia* isolates. All noncarbapenemase producers were negative in the mCNP, except one ESBL-producing *E. coli* isolate. The sensitivity of the mCNP was 95.06% and its speciﬁcity was 94.44%.

## DISCUSSION

Over the past years, carbapenemase producers, mainly Enterobacteriaceae, have spread worldwide, with outbreaks reported in several hospital settings (9, 11, 13). Therefore, rapid and accurate detection of CPE isolates is critical for the treatment and control of infections caused by these microorganisms. Here, we compared five phenotypic tests to detect carbapenemase production among Enterobacteriaceae isolates. Although molecular techniques, such as PCR, remain the gold standard for carbapenemase detection, they are less commonly practiced because of high cost and requirement of a good laboratory facility. PCR-based approaches are not currently available to detect all families of carbapenemases found in CPE. PCR tests for the detection of the major carbapenemase gene families have the potential to quickly detect the presence or absence of any of the five key carbapenemase genes from a single sample.

In the present study, CT-PBA test was detected in both KPC-producing *K. pneumoniae* isolates. The sensitivity and speciﬁcity of this test were 100%. This is in accordance with the report by Dijk et al. (5) that the PBA test is suitable for detecting carbapenemase production in CRE isolates, as the reported sensitivity and specificity were 95% and 99%, respectively. However, the low number of KPC-producing organisms in our study makes it difficult to draw meaningful conclusions from the data presented.

For identification of class B metallo-carbapenemases (MBLs), the sensitivity of the DPA synergy test was 82.61%, with a specificity of 96.22%. The two false-positive results were observed in one ESBL and one OXA-48 producers using the CT-DPA, but eight false-negative results were observed with NDM and NDM-OXA-48-producing isolates. The sensitivity and specificity of the synergy tests using DPA for phenotypic confirmation class B MBL producers were in line with previous reports (5, 14).

In our study, MHT had a low sensitivity and specificity of 80.25% and 83.33%, respectively, with one, seven, and five false-negative results for VIM, NDM, and NDM-OXA-48-producing isolates encountered. Our findings are in agreement with previous studies in which MHT has been found to have poor sensitivity (41–89%) for detection of MBLs (15, 16).

In contrast, with the exception of three isolates, all OXA-48 producers were identiﬁed by this test. It was reported that the sensitivity of MHT was higher for identifying KPC and OXA-48-producing isolates (17). Although the MHT is a simple and inexpensive test for detecting carbapenemases, it had some limitations: the poor sensitivity of the test towards the identification of NDM-producing CRE isolates and poor specificity due to false-positive results in ESBL producers or AmpC overexpression combined with outer membrane porin loss (2, 18). However, the MHT test can still be used in the area, where KPC and/or OXA-48-like carbapenemases are predominant. It should be noted that the weak performance of the MHT for has been well-known for several years; eventually, it has been deleted in CLSI 2018 as a recommended phenotypic method for detecting carbapenemases.

In the current study, the sensitivity of the E-Test MBL was 91.3%, with a specificity of 100%. E-Test MBL detected all NDM, VIM, OXA-48-NDM producers (except for four co-producing NDM-1-OXA-48 isolates). In the case of the E-Test MBL, researchers from Malaysia showed 100% specificity and sensitivity for the detection of carbapenemase in *P. aeruginosa* (19).

The iCarba NP test had a sensitivity of 81.48% and a speciﬁcity of 100% for the detection of carbapenemase producers among the isolates. Class A (KPC) and class B (NDM and VIM) carbapenemases yielded stronger results compared with class D enzymes. The Carba NP test required a maximum of 2 h for monitoring color change, and it detected all KPC (2/2; 100%) and most NDM (13/15; 86.6%) and VIM (2/3; 66.6) carbapenemases in 15 min. Our results are in agreement with some studies in which Carba NP assays exhibited low sensitivity for carbapenemase producers (3, 15). In the present study, 13 isolates of 15 false-negative results missed by the iCarba NP test were revealed by PCR to harbor OXA-48, and other isolates were revealed to harbor both OXA-48 and NDM genes. In agreement with the current study, several studies reported false-negative Carba NP test results for OXA-48-producing isolates (20, 21). The reduced sensitivity of Carba NP was mostly due to the weak carbapenemase activity of OXA-48 isolates.

Using the mCNP, all class A, B, and D carbapenemases were detected (Table 2), with the exception of four OXA-48 producers. These results increased the sensitivity of the test to 95.06%. On the other hand, in this study one isolate showed a false-positive mCNP test in comparison with PCR results that detected no carbapenemase gene after duplicate testing, which conﬁrmed its speciﬁcity of 94.44%. In the current study, the modified Carba NP (mCNP) test is relatively new, although it is similar to the study conducted by Pasteran et al. (22). However, in this study, we also used neutral red in addition to bromothymol blue. In the study of Pasteran et al., 14 Enterobacteriaceae OXA-type producers were examined, of which only 9 (64%) isolates were positive, whereas in our study, 29 of 33 (87.8%) Enterobacteriaceae OXA producers were reported positive. Therefore, this method can be suitable for the detection of Enterobacteriaceae OXA-type producers and other classes of carbapenemase.

Interestingly, unlike iCarba NP test, the results obtained with the mCNP were very promising because most of the OXA-type producers tested positive, which demonstrated the ability of this simpliﬁed mCNP test in detecting OXA-48 carbapenemase. Besides the marked improvement in sensitivity, the color indicator turned yellow much faster than with the mCNP. Regarding the timing of a positive color change by mCNP assay regarding the type of carbapenemase, most of the positive results in this study (41/48), which were mainly KPC, NDM, and OXA-48-NDM carbapenemase producers, produced a color change in the time range between immediate and 15 min. A notable limitation of this study was first, the small number of isolates producing class A carbapenemases (KPC) mainly due to the very rare occurrence of this class of carbapenemase in Iran. Second, the reagent preparation is not commercially available and requires buffer preparation and pH adjustment, as well as false-positive results in the detection of OXA-48.

### Conclusions

In summary, the mCNP test presented the highest sensitivity and, therefore, can be considered the best test to be used as a screening phenotypic methodology. The mCNP test is low-cost to perform and provides rapid and reliable identification of carbapenemase activity, with high sensitivity and specificity. This assay can be carried out routinely in any clinical microbiology laboratory, especially in developing countries, for epidemiological and infection control purposes. However, improving the mCNP test by standardizing could be very important to increase the sensitivity and specificity values.

## Data Availability

The data sets used and analyzed during the current study are available from the corresponding author upon reasonable request.
